# Selection and Characterization of DNA Aptamers Targeting All Four Serotypes of Dengue Viruses

**DOI:** 10.1371/journal.pone.0131240

**Published:** 2015-06-25

**Authors:** Heng-Li Chen, Wen-Hsin Hsiao, Hsiang-Chi Lee, Suh-Chin Wu, Jya-Wei Cheng

**Affiliations:** Institute of Biotechnology and Department of Life Science, National Tsing Hua University, Hsinchu 300, Taiwan; University of Pittsburgh, UNITED STATES

## Abstract

Dengue viruses (DENVs) are members of Flaviviridae family, which are associated with human disease. The envelope (E) protein plays an important role in viral infection. However, there is no effective antibody for clinical treatment due to antibody dependent enhancement of infection. In this study, using Systematic Evolution of Ligands by Exponential Enrichment (SELEX), we demonstrated the first aptamer (S15) that can bind to DENV-2 envelop protein domain III (ED3) with a high binding affinity. S15 was found to form a parallel quadruplex based on Quadfinder prediction, gel mobility assay and circular dichroism studies. Both the quadruplex structure and the sequence on 5’-end were necessary for the binding activity of S15. NMR titration experiments indicated that S15 bound to a highly conserved loop between β_A_ and β_B_ strands of ED3. Moreover, S15 can neutralize the infections by all four serotypes of DENVs. Our result provides a new opportunity in the development of DNA aptamers against DENVs in the future.

## Introduction

Dengue viruses (DENVs) belong to the Flaviviridae family, and contain four serologically and genetically distinct viruses, termed DENV-1, DENV-2, DENV-3 and DENV-4. Like other flaviviruses such as yellow fever virus (YFV), West Nile virus (WNV), Japanese encephalitis virus (JEV) and tick-borne encephalitis virus (TBEV), the rapid and extensive spread of DENV infection has become a major public health concern recently [1].

Flavivirus envelope protein (E protein) is the dominant antigen in eliciting neutralizing antibodies and has an important role in inducing immunologic responses in the infected host [2]. It is also associated with the entry of DENVs into host cells and membrane fusion [3, 4]. E protein is composed of three domains- domain I (ED1), domain II (ED2) and domain III (ED3) [5–7]. ED3 contributes to initial attachment of virus particle to host cell membrane receptors, resulting in further internalization and membrane fusion [3, 8]. Treatments with recombinant ED3 or ED3 neutralizing antibodies appeared to be successful to provide effective protection [9–18], suggesting that ED3-targeting was a feasible strategy to block viral attachment. However, type specific antibodies appeared to enhance the infection when subsequently infected by other serotypes due to antibody dependent enhancement (ADE) [19], and caused more severe diseases such as dengue hemorrhagic fever (DHF) and dengue shock syndrome (DSS) [20, 21]. Therefore, successful treatments should have the ability to simultaneously neutralize all four serotypes of DENVs. Until now, only one tetravalent dengue vaccine has been tested in clinical trial [22].

Systematic Evolution of Ligands by Exponential Enrichment (SELEX) is an important technique to generate short single-strand DNA or RNA aptamers that can efficiently bind to specific targets [23, 24]. A large number of aptamers have been selected for disease-associated targets, including small molecules [25–27], bacteria [28] and whole cells [29]. Efforts were also made towards improving this system [30, 31]. In comparison to antibodies, aptamers have some advantages: 1) they are relatively small and stable, 2) they can be synthesized inexpensively at large scale, and 3) they are unlikely to induce host immune response. Accordingly, aptamer is considered to be a useful alternative for biomedical studies and disease therapy. Moreover, aptamer contains no FcR-binding domain, which is essential for triggering ADE. Here, we present an aptamer targeting DENV-2 ED3. Our results also indicate that this aptamer forms a unique G-quadruplex structure and have the ability to neutralize all four dengue serotypes by binding to a highly conserved region on a loop of ED3.

## Materials and Methods

### Preparation and purification of protein

The DNA fragments encoding residues 288–397 of DENV-2 ED3 were amplified by PCR. PCR products were purified using miniprep kit (Protech), and subsequently digested by NcoI and XhoI and cloned into pETBlue-2 vector (Novagen) which leads to the addition of two non-virus residues, leucine and glutamate, and the His-tag at the C-terminal of ED3. Plasmid was then transformed into E. *coli* strains BL21 (DE3) to allow the over-expression of ED3. The uniform ^15^N-labeled and unlabeled samples were expressed in cells grown in M9 minimal media containing ^15^NH_4_Cl, and Luria-Bertani (LB) broth at 37°C. Cell lysates were prepared using French press and were centrifuged. The pellet was washed by wash buffer (10 mM Tris, pH 7.5, 1 mM EDTA and 1M NaCl) and subsequently denatured using solubilization buffer (100 mM Tris, pH 7.5, 0.2 mM EDTA and 6 M GuHCl) for 16 hours at room temperature. Following centrifugation at 4000 rpm for 30 min, refolding was initiated by rapidly dilution of the denatured sample in refolding buffer (100 mM Tris, pH 7.5, 0.5 M L-arginine and 0.2 mM EDTA). Refolded sample was then dialyzed against purification buffer (20 mM Na_2_HPO_4_, pH 7.5, 300 mM NaCl, 10 mM imidazole, and 100 mM urea) for 2 days with buffer changed each 12 hours. The dialyzed sample was centrifuged at 4000 rpm for 30 min and was subsequently applied to Ni-NTA column according to the protocol provided by the manufacturer (QIAGEN). Column fractions were monitored by 12% SDS page and Coomassie blue staining. Recombinant ED3 was condensed by Amicon microconcentrators (Millipore). ED3 mutants were conducted by site-directed mutagenesis, and were expressed and purified as described above.

### *In vitro* selection of ssDNA aptamers

All Primers, ssDNA library and aptamers were purchased as lyophilized oligonucleotide (Purigo, Taiwan). Prior to use, ssDNA library and aptamers were dissolved in distilled water and were heated at 95°C for 5 min and rapidly cooled on ice. The procedures of SELEX are described as before [30].

DNA library (GGGAAGATCTCGACCAGAAG-N_35_-TATGTGCGTCTACATGGATCCTCA) was used for the selection. In the initial round of selection, an aliquot of one nanomole of ssDNA library was incubated with 2.5 μl ED3-bounded resins (~0.16 nM of recombinant ED3) in 500 μl SELEX buffer (50 mM K_2_HPO_4_, 100 mM NaCl, 0.1 mM EDTA, pH 6.5) at room temperature for 30 min. Unbound ssDNA were removed by washing three times with 1 ml of SELEX buffer. ED3-ssDNA complex were eluted with 10 μl of 500 mM imidazole, and bound ssDNA were amplified using PCR with sense primer (GGGAAGATCTCGACCAGAAG) and 5’-biotinylated antisense primer (TATGTGCGTCTACATGGATCCTCA). PCR products were mixed with 10 μl of Streptavidin Ultralink Resin (PIERCE) for 15 min at room temperature. The resins were washed three times with wash buffer (20 mM Tris, pH 7.5, 1 M NaCl, 1 mM EDTA, and 0.0005% Triton-X 100), and non-biotinylated strand was separated from the immobilized complementary strand by 0.2 M NaOH. The buffer was changed to SELEX buffer using a Centricon (Millipore). In order to avoid the binding of ssDNA to resins, counter-selections were performed after round 3, 6, 9 and 12. 100 μl of ssDNA were incubated with 20 μl of Ni-NTA resins for 30 min at room temperature. After centrifugation, the supernatant was obtained for next round. After 15 rounds of selection, DNA aptamer were cloned into pGEM-T Easy vector (Promega). Plasmids were transformed into E. *coli* strain NovaBlue (Novagen), and fifty colonies were randomly picked for sequencing. Sequence analysis and alignment was performed using BioEdit.

### Site-directed mutagenesis

Plasmid pETBlue-2/ED3 was used as a template. A total of 8 mutants of ED3 (I335A, R345A, Q316G, H317A, G318A, G318P, T319A and I320A) were constructed by QuickChange XL Site-Directed Mutagenesis (Stratagene Co.) according to the instruction. All mutants were confirmed by DNA sequencing.

### Fluorescence quenching

Fluorescence spectra were measured by LS55 Luminescence Spectrophotometer (Perkin-Elmer) in a 1 cm quartz cell, using 10/10 nm slit widths. ED3 (1 μM) was titrated with aptamer to a final ratio of 1:3. The intrinsic fluorescence of ED3 was obtained at 300–400 nm when excited at 280 nm. All measurements were recorded at room temperature, and the log of fluorescence quenching rate was plotted against log of aptamer concentration. The binding constant (k_d_) and the number of binding sites (n) were calculated using Stern-Volmer equation: log((F_0_-F)/F) = Log(1/k_d_)+n*logQ [32], where Q refers to the concentration of aptamer. F_0_ and F refer to the fluorescence intensity in the absence or presence of aptamer.

### Circular dichroism

Parallel G-quadruplex structure was determined using circular dichoism (CD). Folded aptamers (60 μM) were prepared in distilled water and KCl buffer (ranging from 0.1 to 50 mM), respectively. The CD spectra were recorded on an Aviv 202 spectropolarimeter at 1 nm intervals between 320 nm and 200 nm in a 1 mm path cuvette at 25°C. The reported spectrum represented an average of three scans.

### ELISA

Recombinant ED3 (10 μg/mL) was immobilized on an Immuno plate (NUNC) overnight. The plate was washed with PBST and blocked with blocking buffer (PBS containing 3% BSA). Aptamer S15 (100 μM) was diluted 2-fold serially and added into each well (100 μL/well). The plate was incubated at 37°C for 1 hour. Each well was washed three times with PBST, and incubated with 100 μL of Streptavidin-HRP (1:500, GE Healthcare) at 37°C for 1hour. Color was developed by addition of 100 μL of TMB (KPL) for 30 minutes. After the addition of 100 μL of 2N H_2_SO_4_ (100 μL/well), Absorbance measurement at 450 nm was detected using an ELISA spectrophotometer.

### Western blot

Serial-dilutions of recombinant ED3 (4, 8 and 16 ng) were electrophoresed on a 10% SDS page, and then transferred to a PVDF membrane. Biotin-conjugated aptamer S15 (10 μM) and control antibody (anti-His Ab, 1:5000) were used for detecting ED3. The bound aptamer or Ab was then recognized by HRP-streptavidin or anti-mouse Ab followed by ECL detection system (Millipore) according to the manufacture’s instruction.

### NMR spectroscopy

HSQC spectra of ED3 (access number 2JSF) were acquired with a Bruker Avance 600 MHz spectrometer [33]. ^15^N-labeled ED3 was prepared in NMR buffer (10 mM KH_2_PO_4_ and 100 mM NaCl; pH = 6.5) supplemented with 10% D_2_O. ^1^H, ^15^N-HSQC spectra of ED3 were recorded at the ED3-to-S15 molar ratios of 1:0.5, 1:1, 1:2 and 1:3 at 298 K. All spectra were processed and analyzed using the Bruker Topspin and Sparky. Normalized chemical shift changes were calculated by using the equation: δ = [(δH)^2^+(δN/5)^2^]^0.5^.

### Plaque reduction neutralization test

DENVs, including DENV virus serotype 1 HAWAII (access number EU848545), DENV virus serotype 2 New Guinea-C (access number M29095), DENV serotype 3 H-87 (access number M93130), and DENV serotype 4 H241 (access number AY947539) were used to determine the neutralizing activity of S15. Vero E6 target cells were seeded at a density of 5 x 10^5^ cells per 2.5 ml MEM culture medium in each well of a 6-well plate 24 h prior to infection. Approximately 100 plaque-forming units (PFU) of DENV per well were incubated with or without serial dilutions of aptamer in Hank’s Balanced Salt Solution (HBSS) (GIBCO, Grand Island, NY) which contained 0.4% (w/v) bovine albumin fraction V (BSA fraction V) (GIBCO) in a 5% CO_2_ incubator at 37°C for 1 h. Then Virus-aptamer or virus-control mixtures were allowed to infect the confluent monolayer of Vero E6 cells in a 5% CO_2_ incubator at 37°C for an additional 1 h, inoculated mixtures were removed and the cells were washed once with phosphate buffered saline (PBS) and overlaid with overlay medium (1x MEM, 1% methylcellulose, and 10 mM HEPES buffer; pH = 7.4). After 5 days incubation, the plaques were stained with crystal violet dye (1% w/v crystal violet, 0.64% w/v NaCl, and 2% w/v formaldehyde solution) [34]. Calculations of 50% endpoint plaque reduction neutralization titers were made by fitting a dose-response inhibition curve to the results using Graphpad software.

## Results

### Selection of DNA aptamers

**Fig 1:**
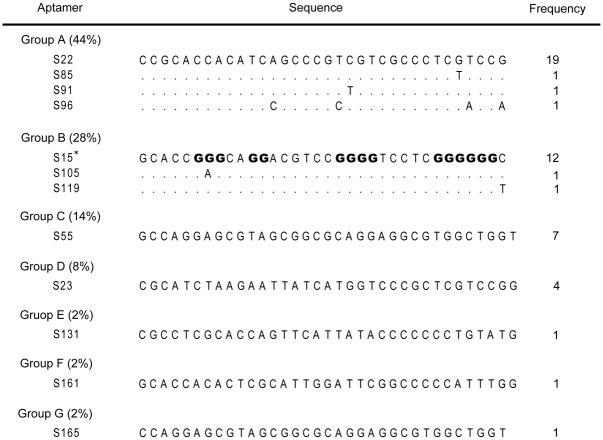
Sequence and frequency of selected aptamers. Selected aptamers were sequenced and were classified into 7 groups.

A single-strained DNA library containing approximately 6×10^14^ molecules was used to select DNA aptamers against DENV-2 ED3. After 15 rounds of selection, we obtained twelve different sequences from fifty clones. According to their sequences, these aptamers were classified into groups A-G (Fig 1). Group A (44%) and group B (28%) were dominant. In contrast, sequences in groups C-G showed much lower frequency. Two dominant aptamer, S22 (frequency = 19/50) and S15 (frequency = 12/50), were selected for the following studies.

### Binding of aptamers to DENV-2 ED3

**Fig 2:**
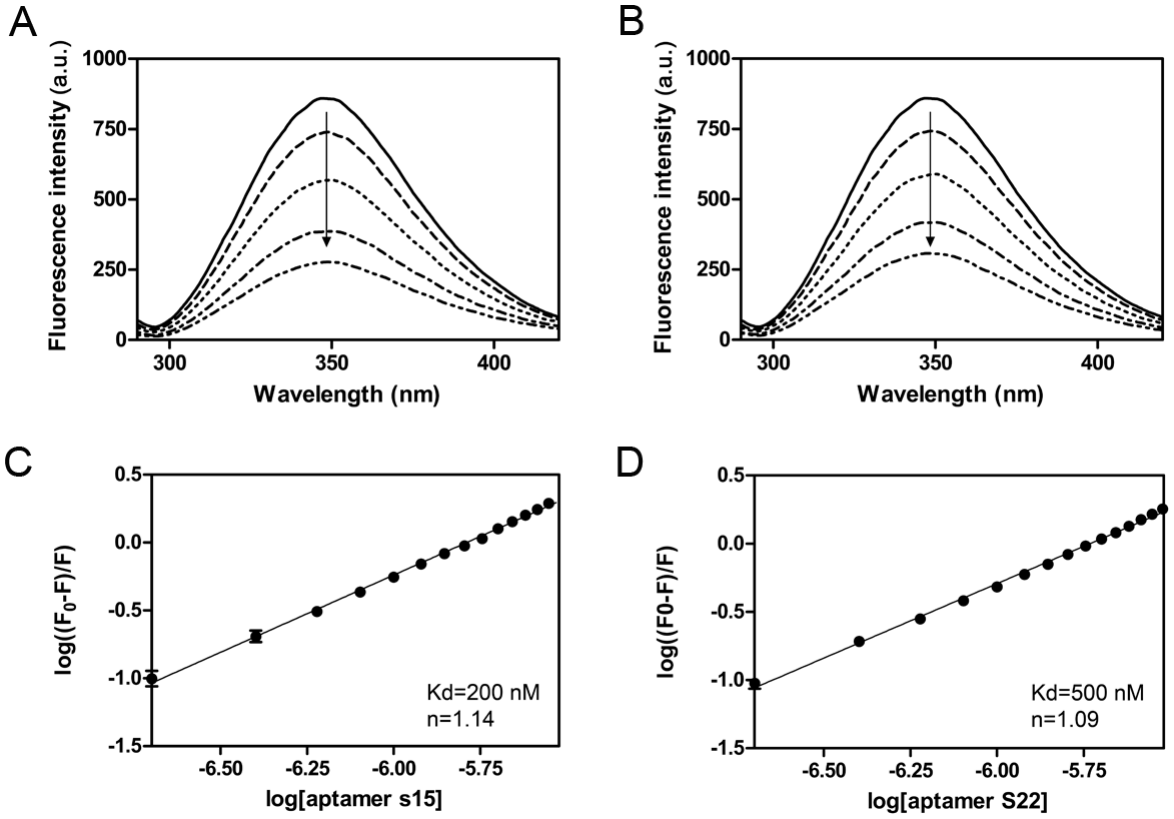
Determination of the binding characteristics of S15 to DENV-2 ED3. Fluorescence Emission Spectra of DENV-2 ED3 titrated with aptamer S15 (A) and S22 (B) in the range from 0 to 3 μM. Addition of aptamer caused significant decreases in the steady-state fluorescence intensity of ED3. The fluorescence quenching (log((F_0_-F)/F) by aptamers S15 (C) or S22 (D) were plotted against aptamer concentration (log(aptamer)). The quenching curve of either S15 or S22 showed a fine linear relationship in the overall concentration range of the aptamers. The correlation coefficient i0.9910 for S15 and is 0.9876 for S22, indicating the static quenching interaction. The dissociation constant and the number of binding sites were calculated by Stern-Volmer equation. The best fit to the data indicated that both of S15 and S22 has one binding site on DENV-2 ED3, and the dissociation constant is 200 nM for S15 and 500 nM for S22. Samples were measured in triplicate and means were shown with standard error.

The interactions between the aptamers (S15 and S22) and DENV-2 ED3 were determined by fluorescence quenching experiment. Tryptophan residue in ED3 gave rise to an emission peak at 350 nm when it was exited at 280 nm. Upon the addition of either aptamer S15 or S22, a significant decrease in fluorescence emission was observed (Fig 2A and 2B). To calculate the binding constant (K_d_) and binding site (n), the plots of fluorescence quenching [log((F_0_-F)/F))] vs. aptamer concentration [log(aptamer)] of aptamers were fitted with Stern-volmer equation, as described in Materials and Methods. The best fit of fluorescence data was found by setting K_d_ = 200 nM and n = 1.14 for aptamer S15. Aptamer S22 had same binding stoichiometry (n = 1.09), but its binding constant was higher than that of aptamer S15 (K_d_ = 500 nM).

### Epitope mapping

**Fig 3:**
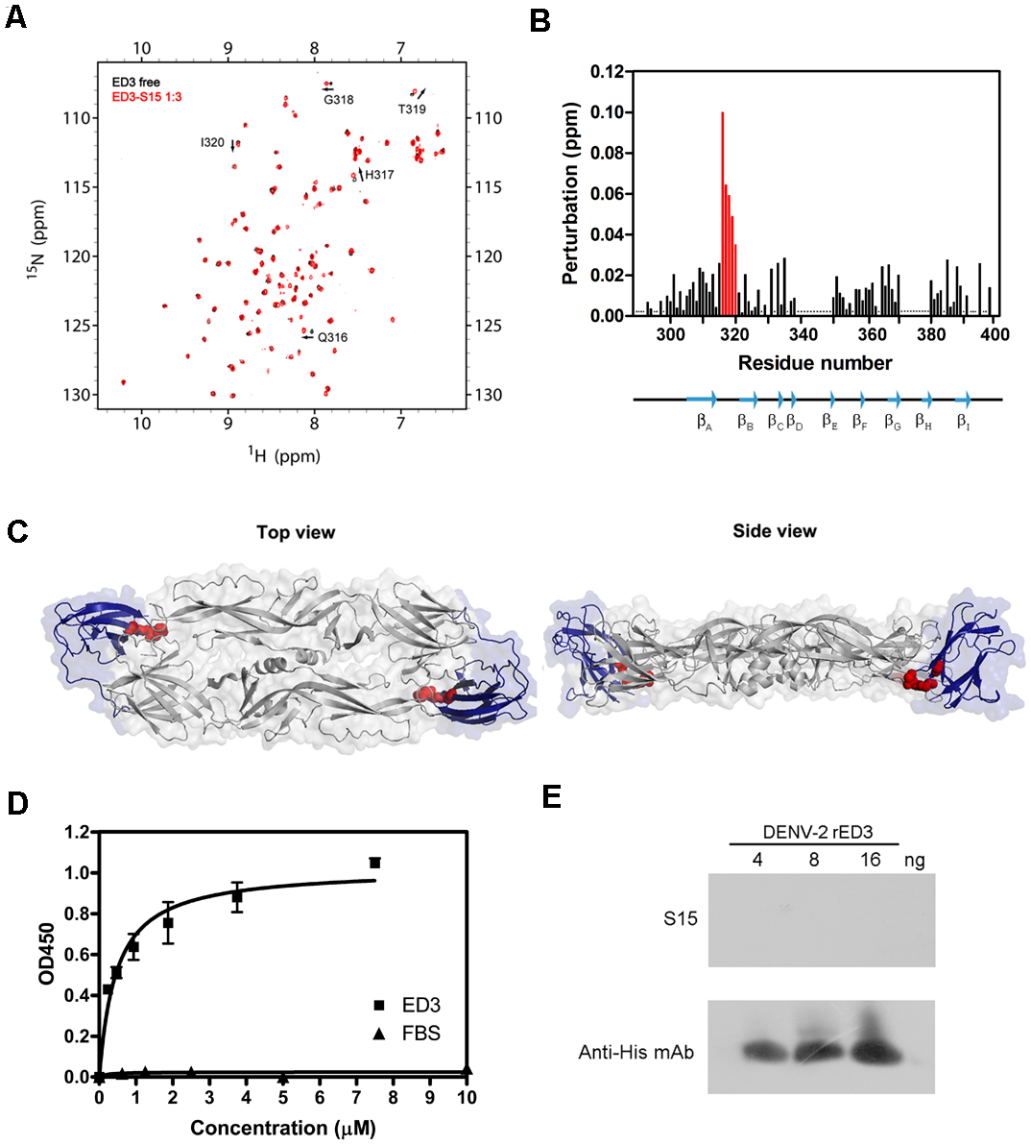
Epitope mapping of aptamer S15 was performed using NMR titration. (A) ED3 was titrated with increasing concentration of aptamer S15. ^1^H-^15^N HSQC spectra of free ED3 is in black and ED3 titrated with S15 is in red. Chemical shift perturbations were observed on residue Q316, H317, G318, T319 and I320. (B) Quantification of chemical shift perturbation of ED3 in the presence of aptamer S15. Asterisks denote residues that were not assigned. It suggested that residues 316–320 are critical for S15 binding. (C) Epitope mapping onto the 3D structure of E protein. Residues 316–320 were mapped on a crystal structure of the DENV-2 E protein dimmer (PDB ID: 1OAN) showed in top view and side view. ED1 and ED2 are in grey, and ED3 are in blue. Residues Q316, H317, G318 and I320 recognized by S15 are in red. (D) Interaction between aptamer S15 and ED3 determined by ELISA. Serial dilutions of S15 were incubated with native ED3. Bound aptamer was determined by indirect ELISA. Samples were measured in triplicate and means were shown with standard error. (D) Interaction between aptamer S15 and ED3 determined by western blotting. Denatured ED3 was detected by western blotting using aptamer S15 and anti-His antibody.

To identify the epitope on ED3, we performed a HSQC titration experiment with aptamer S15. The ED3 backbone NOE’s of ED3 remained approximately as that of free ED3 in the presence of aptamer S15 (Fig 3A), suggesting that the overall structure was not influenced by aptamer S15. When the concentration of aptamer S15 increased, we observed significant perturbations on the chemical shift of residue Q316, H317, G318, T319 and I320 (Fig 3A and 3B). According to three dimensional structure of ED3, we found that residue 316–320 were located on the loop between β_A_ and β_B_ strand (Fig 3C).

Site-directed mutagenesis was used to alter these residues to confirm their essential role for aptamer S15 binding. As shown in Table 1, Q316G and I320A mutations resulted in more than 4-fold decrease in the binding (K_d_ = 860 and 867 nM). G318A mutation showed less effect on S15 binding. However, when G318 was substituted with proline [35], it caused a 5-fold decrease in the binding (K_d_ = 1008 nM). Proline has a cyclic structure. The G318P mutation may reduce the flexibility of the epitope loop, hence affects the binding. Similar decrease of binding was found for the H317A mutation (K_d_ = 1027 nM). In contrast, T319A mutation showed tiny effect on the binding (K_d_ = 378 nM) demonstrating that residue T319 may be less important for aptamer S15 binding. Mutations on residue I335 and R345 that were distant from the epitope showed no significant effect on S15 binding (K_d_ = 279 and 190 nM).

**Table 1:** Dissociation constant (K_d_) and stoichiometry (n) of S15 to wild type ED3 and mutants.

rED3	S15	
	Dissociation constant	Number of binding site (n)
WT	200	1.182
I335A	279	1.154
R345A	190	1.181
Q316G	860	1.099
H317A	1027	1.074
G318A	606	1.095
G318P	1008	1.079
T319A	378	1.139
I320A	867	1.094

ELISA binding assays were conducted to determine the specificity and cross-reaction of the aptamer S15. The results showed that S15 can bind to ED3 in a dose-dependent manner. In contrast, when using fetal bovine serum (FBS) as the target, no cross-reaction was observed (Fig 3D). In addition, we found that S15 was not able to bind denatured ED3 in the western blot (Fig 3E). This result suggested that S15 binds to a conformation epitope instead of a linear epitope [36].

### *In vitro* neutralization of DENVs

**Fig 4:**
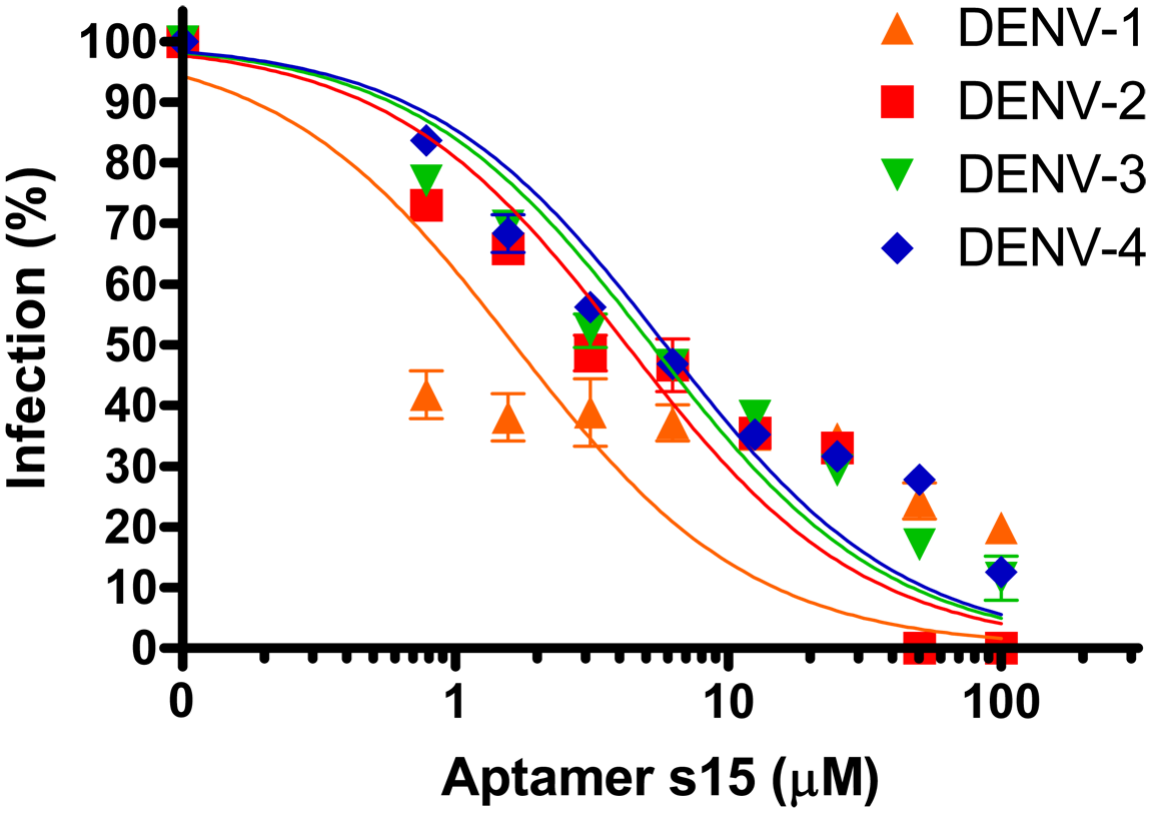
Neutralization activity of S15 against all four DENV. Following incubation with various concentrations of aptamer S15 (0–100 μM), DENV HAWAII, DENV-2 NGC, DENV-3 H-87, and DENV-4 814669 were further incubated with Vero E6 cells, respectively. The infection was determined by PRNT assay. Samples were measured in triplicate and means were shown with standard error.

**Fig 5:**
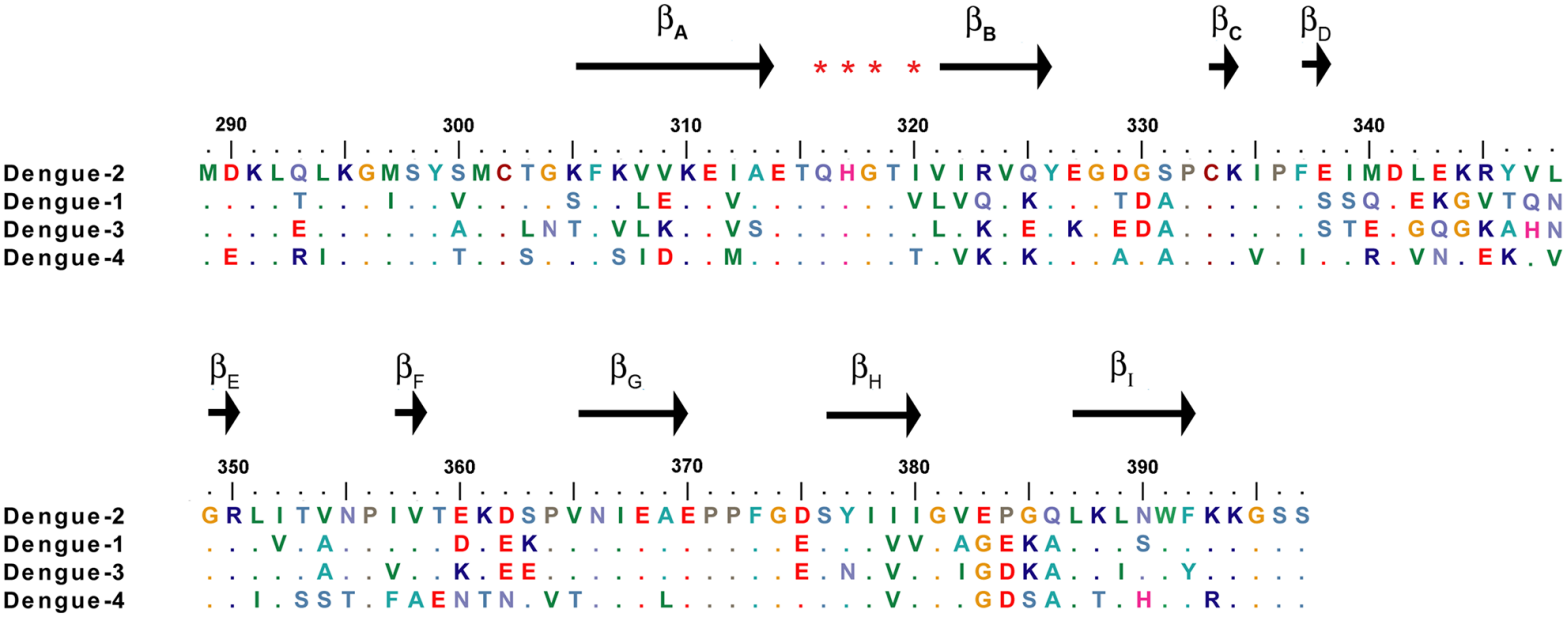
Structure-based sequence alignment of the ED3 of four serotypes of DENVs. Identical amino acids among all four DENV were shown in dot. Asterisks denote residues that have significant chemical shift upon the addition of S15.

Aptamer S15 was able to recognize DENV-2 virion as well as ED3 (data not shown), although the epitope appeared to be poorly accessible in the native virus structure [37]. We performed a PRNT assay to determine the neutralization activity against DENVs. The results showed that the infection of DENV-2 NGC was completely neutralized by 100 μM S15 (Fig 4). The IC_50_ was 4.2 μM. Moreover, using structure-based sequence alignment we found that residues (Q316, H317 and G318) on the binding epitope for aptamer S15 are identical (Fig 5). We therefore tested the neutralization activity against DENV-1 HAWAII, DENV-3 H-87 and DENV-4 H241. The IC_50_ values of the other three dengue serotypes are in similar range with DENV-2 NGC (IC_50-DENV-1_ = 1.7 μM, IC_50-DENV-3_ = 5.2 μM, IC_50-DENV-4_ = 5.8 μM). However, only DENV-2 NGC can be completely neutralized by S15 at 100 μM.

### Functional and structural characterization of aptamer S15

**Fig 6:**
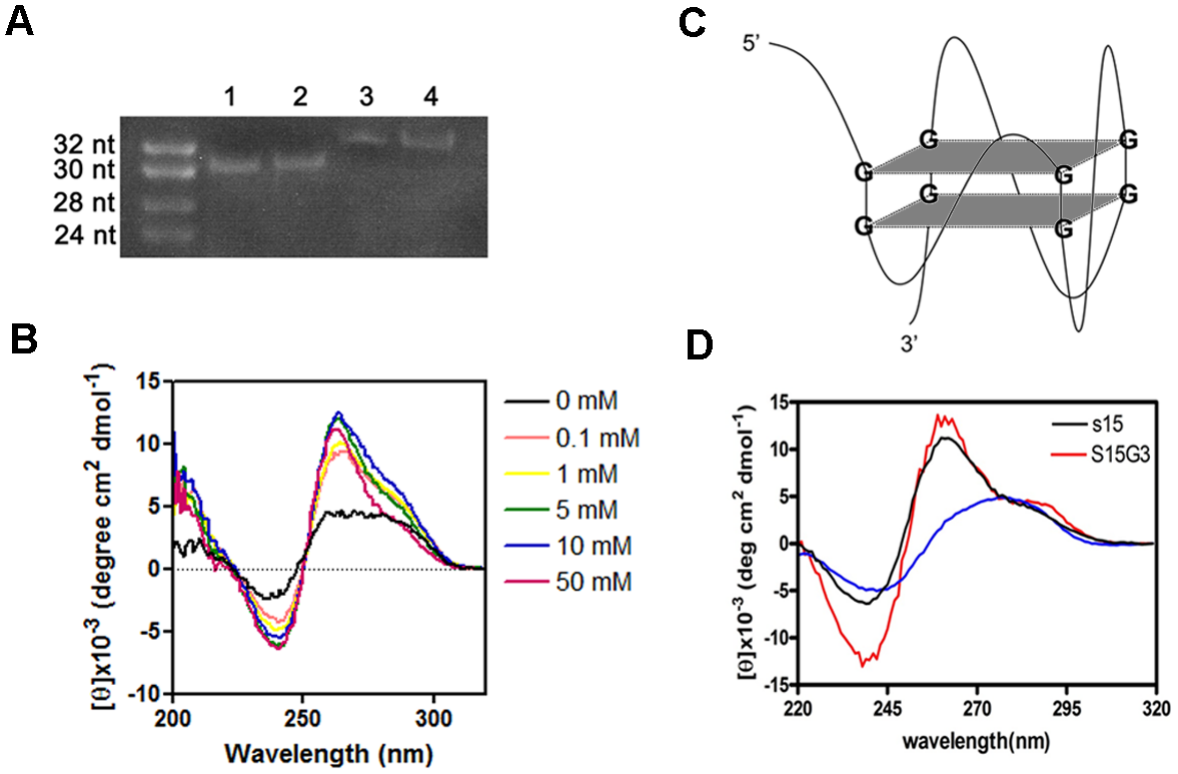
Analysis of quadruplex structure. (A) Native gel was used to determine the folding of S15. Lane 1 and lane 2 are S15 prepared in distilled water and 100 mM KCl. Lane 3 and lane 4 are other 34 mer linear nucleotides prepared in distilled water and 100 mM KCl. Gel mobility of aptamer S15 was faster than linear nucleotides which has same length, suggesting that S15 folds into specific structure as monomer. (B) CD spectra of aptamer S15 in varying concentration of KCl (0 to 50 mM). S15 in 5 mM of KCl showed a negative peak at 240 nm and a positive peak at 264 nm, indicating the formation of parallel quadruplex structure. (C) Illustration of parallel quadruplex structure of aptamer S15 with two G·G·G·G tetrad and external loops. (D) Comparison of CD spectra of aptamer S15, aptamer S15G2 and S15G3. It suggested that aptamer S15 and aptamer S15G3 had the same parallel quadruplex structure.

In the native gel electrophoresis, we found that aptamer S15, in either water or 100 mM KCl buffer, had a faster mobility than other 34-mer oligonucleotide (Fig 6A). Aptamer S15 contained 17 guanines separated into four guanine-rich regions. According to the prediction by QuadFinder (G stretch: 2–5, N stretch: 1–10), aptamer S15 had a G_2_L_1-10_ profile between G6 and G33, and was able to fold into a G-quadruplex structure composed of two G·G·G·G tetrads and external loops (Fig 1). The result was confirmed by QGRS Mapper (G-score = 32) [38]. Furthermore, we found that aptamer S15 exhibited one negative band at 240 nm and two positive bands at 264 and 290 nm in the CD spectrum (Fig 6B). When we increased the concentration of potassium ion to 5 mM, a minimum band at 240 nm, a maximum band at 264 nm, and a shoulder at 290 nm were induced. The results indicated that aptamer S15 can fold into a parallel G-quadruplex as illustrated in Fig 6C.

To investigate the essential part of aptamer S15 for its neutralization activity, two aptamers (S15G2 and S15G3) derived from aptamer S15 were designed. Aptamer S15G2 contained eight guanines separated into four clusters. However, CD spectrum of aptamer S15G2 showed no similarity to aptamer S15 (S1 Fig). In contrast, aptamer S15G3 which contained twelve guanines in four clusters showed a very similar CD spectrum to aptamer S15 (Fig 6D). It also exhibited a similar binding affinity to aptamer S15 (Table 2). We also synthesized several aptamers (S15G3L1A, S15G3L2A, S15G3L3A and S15G3L123A), of which the internal loops were substituted by alanine. Structures of these aptamers were very similar to aptamer S15 by CD spectrum (S1 Fig) As shown in Table 2, aptamers S15G3L1A, S15G3L2A, S15G3L3A and S15G3L123A exhibited similar structure and binding affinity compared with aptamer S15G3 (Kd = 230, 260, 200 and 266.5 nM). However, when we further deleted the sequence at both end (GCACC at 5’ end and C at 3’ end), the binding activities of aptamer S15G3L12AdT, S15G3L13AdT, S15G3L23AdT and S15G3L123AdT were dramatically dropped (Kd = 1.1, 1.422, 3.46 and 2.78 μM). The results suggested that the terminal sequences were involved in the interaction between aptamer S15 and ED3 instead of internal loops. Furthermore, aptamer 5Ts (Kd = 600 nM) and 5T-polyT (Kd = 525 nM) that contained GCACC sequence were found to show stronger binding activity compared to the polyT control (Kd = 1426 nM). The results suggested that both GCACC sequence and G-quadruplex structure were essential for the neutralization activity.

**Table 2:** Sequence, dissociation constant and stoichiometry of oligonucleotides derived from S15.

Aptamer	Sequence (5' to 3')	Kd (nM)	N
S15G3	GCACC**GGG**CA**GGG**ACGTCC**GGG**TCCTC**GGG**C	292	1.137
S15G3L123AdT	**GGG**A**GGG**A**GGG**A**GGG**	2780	1.028
S15G3L23AdT	**GGG**CA**GGG**A**GGG**A**GGG**	3460	1.024
S15G3L13AdT	**GGG**A**GGG**ACGTCC**GGG**A**GGG**	1422	1.062
S15G3L12AdT	**GGG**A**GGG**A**GGG**TCCTC**GGG**	1100	1.111
S15G3L123A	GCACC**GGG**A**GGG**A**GGG**A**GGG**C	266.5	1.173
S15G3L1A	GCACC**GGG**A**GGG**ACGTCC**GGG**TCCTC**GGG**C	230	1.171
S15G3L2A	GCACC**GGG**CA**GGG**A**GGG**TCCTC**GGG**C	260	1.164
S15G3L3A	GCACC**GGG**CA**GGG**ACGTCC**GGG**A**GGG**C	200	1.213
5Ts	GCACC**GGG**A**GGG**	600	1.15
5T-polyT	GCACCTTTTTTTTTTTTTTTC	525	1.127
polyT	TTTTTTTTTTTTTTTTTTTTT	1426	1.06

## Discussion

DENV infection has become an emerging disease. Previously, ED3 has been used to raise antibodies for the treatment of infections by DENVs. Those antibodies have been shown to exhibit different potentials to neutralize the infection [39–41]. Among them, type-specific and subcomplex-specific antibodies commonly target the lateral surface and β_A_ strand, of which residues are not identical among all four DENVs. They, therefore, failed to treat infections by DENVs due to ADE. In contrast, antibodies (5A2-7, 13D 4–1, E111, and E114) that targeted a highly conserved loop between β_A_ and β_B_ strands exhibited cross-reactive neutralization activity. However, 3D structure of E protein revealed that the loop appeared to have limited accessibility on the mature virion, leading to the poor neutralizing activity of cross-reactive antibodies (IC_50_ > 100 μg/ml). Since the first aptamer based drug, mercugen, was approved by FDA in 1999 [42], aptamers have been shown to possess enormous function as antibodies. Compared to antibody, aptamer has an advantage of lacking FcR-binding motif, leading to a possibility to treat DENVs without causing ADE. In this study, we successfully selected the first aptamer against DENVs. We found that aptamer S15 binds to the conserved loop between β_A_ and β_B_ strands of ED3. In comparison to antibodies, aptamer S15 has smaller size (~10KDa), which may be helpful for improving the interaction between S15 and the poorly accessible loop. Significantly, it exhibited a neutralization activity (IC_50_ < 60 μg/ml) stronger than cross-reactive antibodies (Fig 4). Although S15 can neutralize all four DENVs via binding to a conserved epitope, the antiviral efficacy of S15 was limited against heterologous serotypes. This may be due to the differences of electrostatic surfaces of all dengue serotype ED3. By comparing the top view of all dengue serotype ED3, we found that the electrostatic surface of ED3 of DENV-1 (PDB ID: 3IRC), DENV-3 (PDB ID: 1UZG) and DENV-4 (PDB ID: 2H0P) were slightly more negative than that of DENV-2 (PDB ID: 2JSF). Hence, the electrostatic repulsion may limit the interactions between S15 and ED3 of DENV-1, DENV-3 and DENV-4.

Guanine-rich aptamers have been shown to form a variety of G-quadruplex structures containing stacked G·G·G·G tetrads in the presence of potassium [43–47]. Aptamers against HIV-1 integrase [44], thrombin [48], and bovine prion protein [49] were found to form unique G-quadruplex structures. Similarly, our data including computer prediction, gel mobility assay and CD spectrum suggested that aptamer S15 can fold into a parallel G-quadruplex structure, which appeared to be more stable in the presence of 5 mM potassium ion (Fig 6A, 6B and 6C). To identify the essential sequence for the binding, we designed aptamer S15G3 by rearranging the positions of guanines. Adenosine substitutions of internal loops (S15G3L1A, S15G3L2A, S15G3L3A and S15G3L123A) showed that internal loops were not involved in the binding, but the GCACC sequence on 5’-end was involved (Table 2). Titration of aptamer S15G3L123A, an aptamer containing no internal loops, also caused chemical shift perturbations on residues Q316, H317, G318 and T319 (S2 Fig). It provided the evidence that rearrangement of guanine positions would not influence the structure and function of aptamer S15. Although GCACC sequence can bind to ED3, no effective neutralization activity of aptamer 5Ts was observed by plaque assay (S3 Fig). We therefore speculated that the G-quadruplex structure may be important for blocking the attachment of E protein to host receptor. ELISA and western blot data revealed that S15 forms a specific binding to the ED3 of DENVs (Fig 3D and 3E). However, the structure of S15/ED3 complex remains to be determined to understand the detail interactions.

Overall, our present study provides the first aptamer with antiviral activity against four serotypes of DENV. Our results also indicate that residues on the loop between β_A_ and β_B_ strands of DENV ED3 are of great importance for neutralizing the four dengue serotypes. Our result provides a new opportunity in the development of DNA aptamers against DENVs in the future.

## Supporting Information

S1 Fig CD spectra of the aptamers studied in Table 2. (TIF)

S2 Fig ^1^H-^15^N HSQC spectra of free ED3 is in black and ED3 titrated with S15G3L123A is in red. (TIF)

S3 Fig No effective neutralization activity of aptamer 5Ts was observed by plaque assay. (TIF)
