# Supplementary figures and images for: Selection and Characterization of DNA Aptamers Targeting All Four Serotypes of Dengue Viruses
Source: PLoS One. 2015 Jun 25;10(6):e0131240. doi: 10.1371/journal.pone.0131240 (PMC4482433; doi:10.1371/journal.pone.0131240)

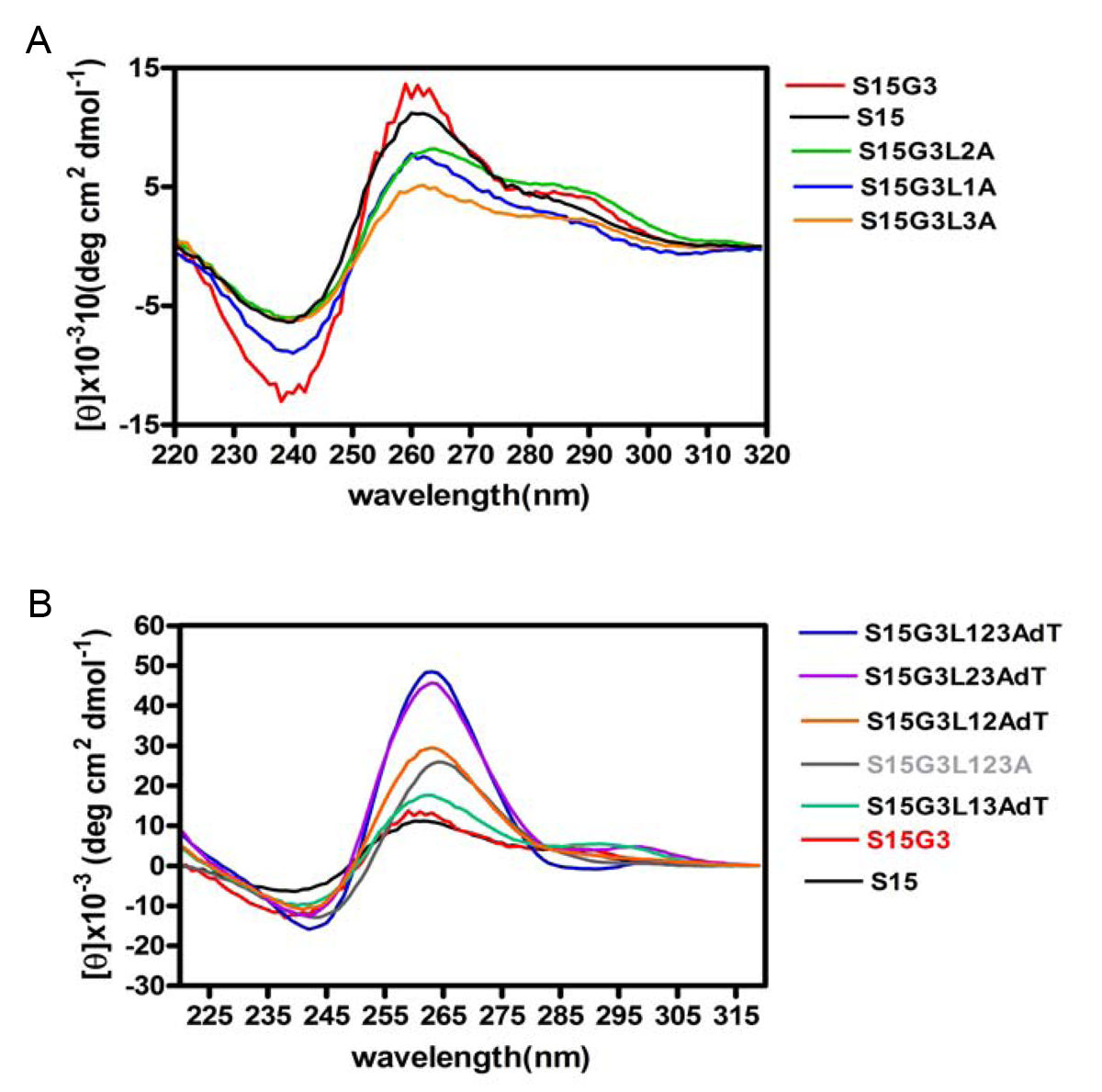

Supplement: S1 Fig — (TIF) [file pone.0131240.s001.tif]

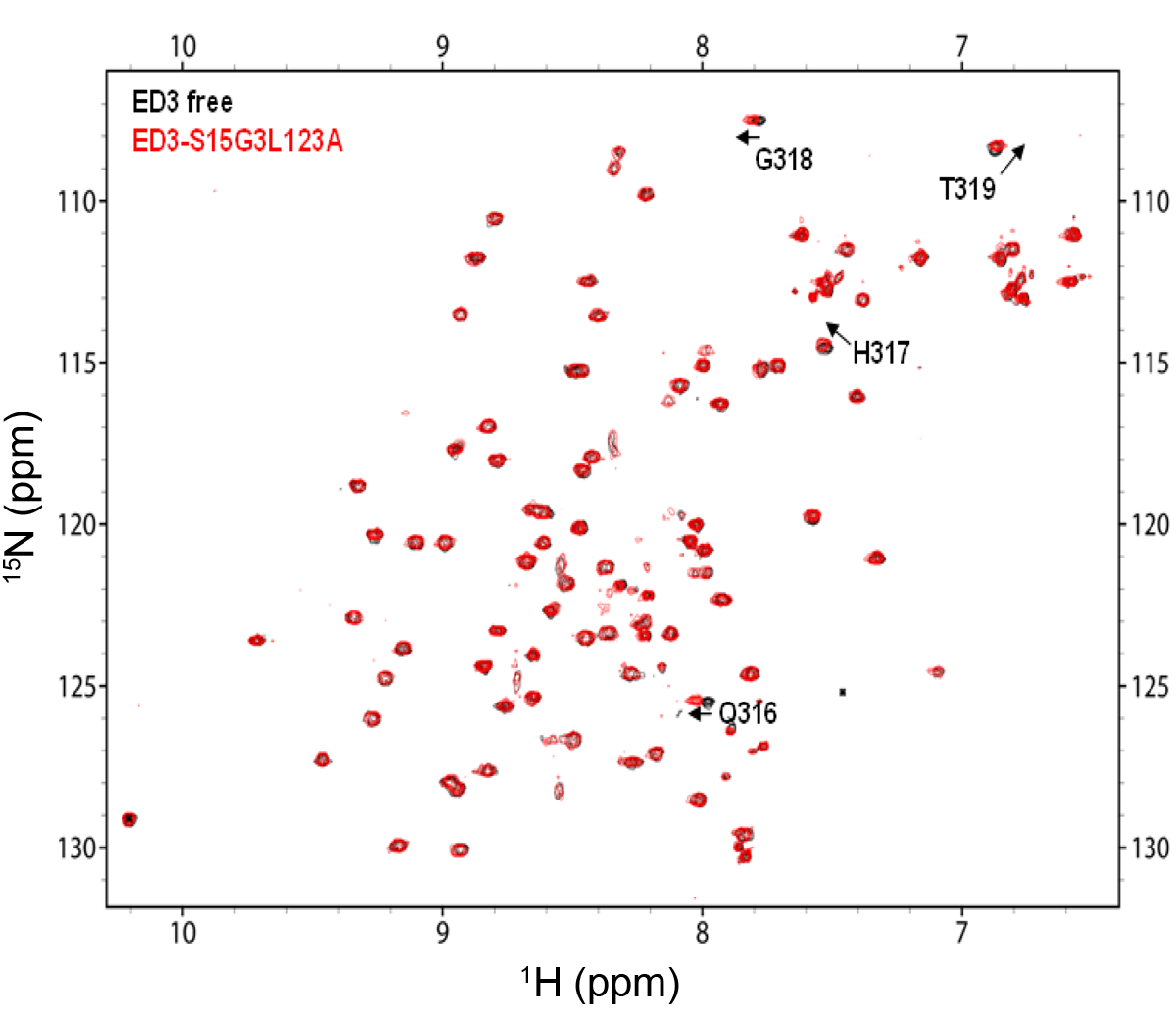

Supplement: S2 Fig — (TIF) [file pone.0131240.s002.tif]

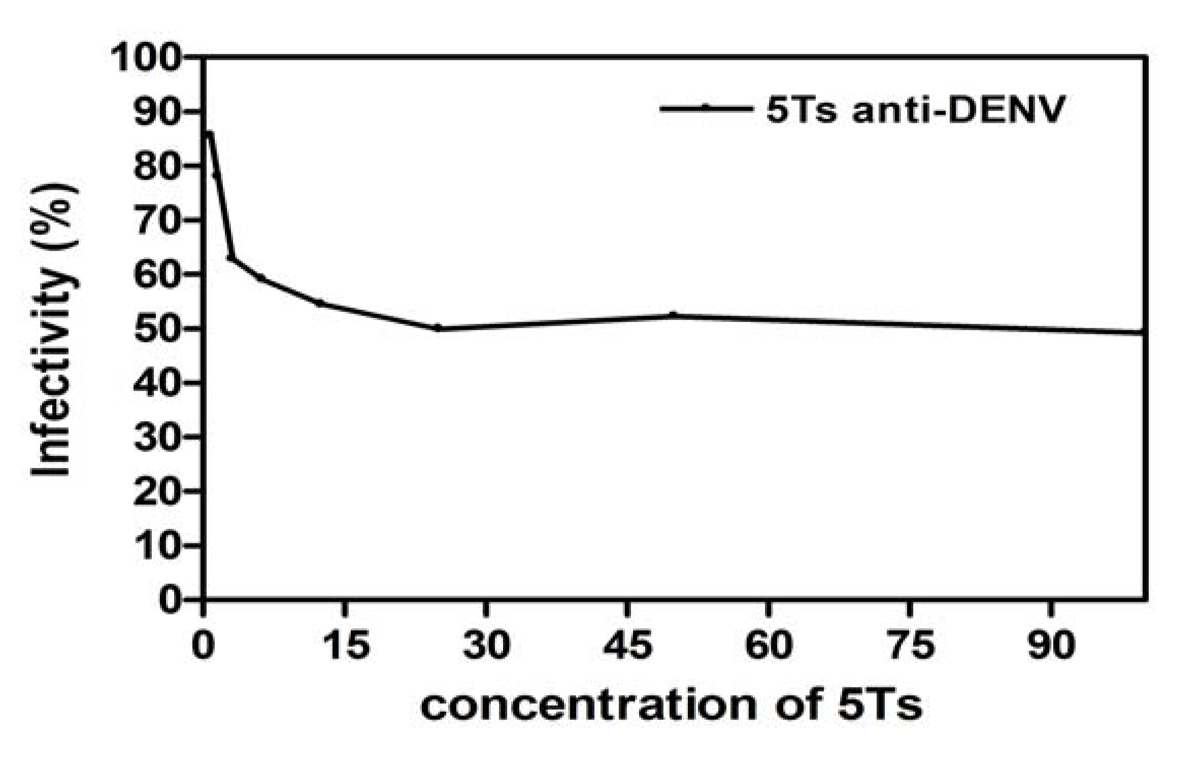

Supplement: S3 Fig — (TIF) [file pone.0131240.s003.tif]
